# Fluorescence-based tracing of transplanted intestinal epithelial cells using confocal laser endomicroscopy

**DOI:** 10.1186/s13287-019-1246-5

**Published:** 2019-05-27

**Authors:** Fredrik Bergenheim, Jakob B. Seidelin, Marianne Terndrup Pedersen, Benjamin E. Mead, Kim B. Jensen, Jeffrey M. Karp, Ole Haagen Nielsen

**Affiliations:** 1 Department of Gastroenterology, Herlev Hospital, University of Copenhagen, 2730 Herlev, Denmark; 2 Biotech Research and Innovation Center (BRIC), University of Copenhagen, DK-2200 Copenhagen, Denmark; 3 Broad Institute of Massachusetts, Institute of Technology and Harvard University, Cambridge, MA 02139 USA; 4 Ragon Institute of Massachusetts General Hospital, Massachusetts Institute of Technology and Harvard University, Cambridge, MA 02139 USA; 5 Koch Institute for Integrative Cancer Research, Massachusetts Institute of Technology, Cambridge, MA 02139 USA; 6 Institute for Medical Engineering and Science (IMES), Massachusetts Institute of Technology, Cambridge, MA USA; 7 Novo Nordisk Foundation Center for Stem Cell Biology (DanStem), Faculty of Medical and Health, University of Copenhagen, DK-2200 Copenhagen, Denmark; 8 Engineering in Medicine, Department of Medicine, Center for Nanomedicine, Brigham and Women’s Hospital, Harvard Medical School, 02115 Boston, MA USA; 9 Harvard-Massachusetts Institute of Technology Division of Health Sciences and Technology, Massachusetts Institute of Technology, 02139 Cambridge, MA USA

**Keywords:** Cell labeling, Confocal laser endomicroscopy, Colitis, Fluorescent dyes, Intestinal organoids, Intestinal stem cells, Nanoparticles, Transplantation

## Abstract

**Background:**

Intestinal stem cell transplantation has been shown to promote mucosal healing and to engender fully functional epithelium in experimental colitis. Hence, stem cell therapies may provide an innovative approach to accomplish mucosal healing in patients with debilitating conditions such as inflammatory bowel disease. However, an approach to label and trace transplanted cells, in order to assess engraftment efficiency and to monitor wound healing, is a key hurdle to overcome prior to initiating human studies. Genetic engineering is commonly employed in animal studies, but may be problematic in humans due to potential off-target and long-term adverse effects.

**Methods:**

We investigated the applicability of a panel of fluorescent dyes and nanoparticles to label intestinal organoids for visualization using the clinically approved imaging modality, confocal laser endomicroscopy (CLE). Staining homogeneity, durability, cell viability, differentiation capacity, and organoid forming efficiency were evaluated, together with visualization of labeled organoids in vitro and ex vivo using CLE.

**Results:**

5-Chloromethylfluorescein diacetate (CMFDA) proved to be suitable as it efficiently stained all organoids without transfer to unstained organoids in co-cultures. No noticeable adverse effects on viability, organoid growth, or stem cell differentiation capacity were observed, although single-cell reseeding revealed a dose-dependent reduction in organoid forming efficiency. Labeled organoids were easily identified in vitro using CLE for a duration of at least 3 days and could additionally be detected ex vivo following transplantation into murine experimental colitis.

**Conclusions:**

It is highly feasible to use fluorescent dye-based labeling in combination with CLE to trace intestinal organoids following transplantation to confirm implantation at the intestinal target site.

**Electronic supplementary material:**

The online version of this article (10.1186/s13287-019-1246-5) contains supplementary material, which is available to authorized users.

## Background

Intestinal stem cells situated at the base of crypts of Lieberkühn generate progeny that replace resident cells, which are shed from the tip of villi as part of the homeostatic process*.* These stem cells can in vitro be propagated as organoids [1], and orthotopic transplantation in murine models of mucosal injury has revealed that intestinal organoids are able to spontaneously attach and integrate into the damaged epithelium [2–5], thereby accelerating the healing process with subsequent improvement in weight gain [3]. This suggests that transplantation of intestinal stem cell might be applicable in humans to actively promote mucosal healing [6] and could potentially be used to treat a wide range of gastrointestinal disorders, including inflammatory bowel disease, in which mucosal healing is a pivotal treatment goal [7, 8] and the most important predictor of clinical remission [9–11]. A method to trace transplanted cells in vivo is, however, essential to assess engraftment efficiency and to monitor wound healing, especially in the preclinical phase.

Confocal laser endomicroscopy (CLE) is an established and clinically approved endoscopic modality permitting high-resolution and real-time imaging of fluorophores in distinct spatial planes [12, 13]. Although fluorescence has limited penetration depth, CLE is able to get very close to the mucosa, thereby mitigating such limitations. At the same time, CLE allows for endoscopic evaluation of the intestinal wound surface [12, 13], which in turn is not possible using other labeling methods such as single-photon emission computed tomography, positron emission tomography, or magnetic resonance imaging (MRI).

In previous murine studies of intestinal transplantation [2–5], cells were genetically engineered to express green fluorescent protein. Although this constitutes a long-lasting labeling technique, such a strategy may cause off-target genetic alterations with unknown long-term adverse effects in humans [14]. Therefore, we investigated the applicability of a panel of readily available fluorescent dyes and nanoparticles using intestinal organoids as well as orthotopic transplantation in an experimental colitis model. The dyes included fluorescein, 5-chloromethylfluorescein diacetate (CMFDA), a carbocyanine-based dye, along with an inert membrane permeable dye. Additionally, two different types of nanoparticles were studied (quantum dots and dye-loaded poly lactic-co-glycolic acid (PLGA) nanoparticles), which both have been used to track and manipulate other cell types [15–17]. The dyes and nanoparticles were chosen based on an expected retention time of at least 24 h, and selection was limited to dyes and particles emitting in the green spectrum, because clinically approved CLE endoscopes are equipped solely with a 488-nm excitation laser.

The different labeling techniques were evaluated in terms of homogeneity, transfer to adjacent unlabeled cells, and effects on cell viability and function, as well as fluorescent signal intensity and durability. The aim of the study was to investigate the feasibility of fluorescent-based longitudinal tracing of intestinal epithelial cells using CLE.

## Methods

### Isolation of colonic crypts and establishment of organoid cultures

Human colonic biopsies were harvested from healthy control subjects and from patients with quiescent ulcerative colitis (endoscopic Mayo subscore 0), as described in Li et al. [18]. In summary, samples were washed in cold Gibco^TM^ Dulbecco’s phosphate-buffered saline (DPBS; Thermo Fisher Scientific, Waltham, MA, USA), and cell dissociation was promoted using ethylenediaminetetraacetic acid (EDTA (8 mM), Thermo Fisher Scientific) on a rocking platform at 5 °C for 1 h. Crypts were released through forceful shaking and subsequently homogenized in diluted growth factor reduced Matrigel^®^ Matrix (Corning Inc., Corning, NY, USA), and cultured in standard culture medium [18, 19]. Organoids were initially cultured for two passages, before experimental setups were initiated.

Murine colonic organoids were established from ROSA mT/mG mice (stock.nr. 007576, Jackson Laboratory, Bar Harbor, ME, USA) essentially as described above using EDTA (10 mM) supplemented with dithiothreitol (80 μg/ml) (Bio-Rad Laboratories, Hercules, CA, USA) to release the crypts from the tissue fragments. The murine organoids were cultured in basal medium supplemented with recombinant R-spondin 1 (500 ng/ml) (R&D systems, MN, USA), Gibco™ recombinant murine EGF (50 ng/ml) (Thermo Fisher Scientific), recombinant murine Noggin (100 ng/ml) (Peprotech, Rocky Hill, NJ, USA), Gibco™ B-27 without vitamin A (Thermo Fisher Scientific), Gibco™ N-2 supplement, Nicotinamide (10 mM) (Sigma-Aldrich, St. Louis, MO, USA), CHIR99021 (Calbiochem, San Diego, CA, USA), PGE_2_ (2.5 μM) (Sigma-Aldrich), and valproic acid (1 mM) (Sigma-Aldrich).

### Cell staining protocols

Organoids were stained in accordance with instructions provided by the manufacturers, as well as previously published protocols. All staining experiments were performed in triplicates, and at least three separate experiments were performed if not otherwise specified. In cases where successful staining were obtained, each culture well was imaged at five representative locations to assess staining efficiency.

#### Fluorescein

Organoids were suspended and incubated at 37 °C in fluorescein-laden culture medium for up to 6 h (fluorescein sodium salt (40 μM) [20] (Sigma-Aldrich). Organoids were subsequently washed three times with basal medium before performing fluorescence imaging.

#### Membrane permeable and inert dye

The culture medium was supplemented with an inert and membrane permeable green fluorescent dye (40–400 μg/ml) (Phosphorex, Hopkington, MA, USA) for up to 24 h, or cells were stained in suspension for up to 6 h. Three washing procedures were performed before fluorescence imaging.

#### CMFDA

Organoids were suspended in a basal medium solution containing 5, 15 or 25 μM of green CMFDA (*CellTracker*™, Thermo Fisher Scientific) and incubated at 37 °C for 45 min. Three consecutive washes were then performed before imaging.

#### Carbocyanine-based cytoplasmic membrane dye

Organoids were stained in suspension at 37 °C in 1 ml of basal medium supplemented with 5 μl of *CellBrite*™ green cytoplasmic membrane dye (Biotium, Fremont, CA, USA) for up to 40 min. Cells were repeatedly washed with basal medium before fluorescence observation.

#### Quantum dots

A 2–15 nM labeling solution of *Qtracker*® 525 cell labeling kit (Thermo Fisher Scientific) was prepared by pre-mixing its two components (nanocrystals and a custom carrier). The solution was incubated at 37 °C for 5 min, after which 200 μl of culture medium containing suspended organoids or single cells was added to the labeling solution. The mixture was incubated at 37 °C for up to 1 h. Cells were afterwards washed twice and imaged. Staining was similarly pursued using the same staining protocol after single-cell dissociation.

#### Fluorescent PLGA nanoparticles

Green fluorescent PLGA nanoparticles loaded with a BOPIDY-FL dye (Thermo Fisher Scientific) and with an average diameter of 150.6 nm (SD = 5.3 nm) were produced by single emulsion solvent evaporation techniques [21] with a lactide to glycolide ratio of 1:1 and a MW of 30.000 (Lactel Absorbable Polymers, Birmingham, AL, USA).

The surface charge of the particles was modified using poly-l-lysine (PLL) solution. In brief, 5 mg of lyophilized particles was resuspended in PLL solution (0.01%, *w*/*v*) (Sigma-Aldrich) and the suspension was shaken at 37 °C for approximately 2 h [21].

The ζ-potential of the PLGA particles were measured in distilled water using a Nano-ZS90 Zetasizer (Malvern Instruments Ltd., Malvern, UK). The ζ-potential of uncoated and PLL-coated PLGA nanoparticles was − 21.4 mV (SD = 4.31) and + 13.2 mV (SD = 3.58), respectively. Uncoated or PLL-coated particles were reconstituted in basal medium and were briefly probe-sonicated with a Branson Digital Sonifier 450 (Branson Ultrasonics, Dietzenbach, Germany), and the PLGA-basal medium solution was subsequently used to prepare a particle-laden culture medium with a PLGA concentration of 0.1 mg/ml [21]. The culture medium was added to each culture well for a duration of 24 h. Alternatively, particles were mixed into the diluted Matrigel^®^ solution to minimize the diffusion distance [22]. Labeling of organoids as well as single cells were similarly attempted using a particle-laden suspension for 4–6 h at 37 °C.

### Evaluation of intercellular transfer

The utility of the staining techniques by which organoids were effectively labeled were further assessed in terms of intercellular transfer. Organoids were stained with green CMFDA (15 μM), carbocyanine-based cytoplasmic membrane dye (5 μl/ml), or the green membrane permeable inert dye (40 μg/ml). After completion of three consecutive washing steps, stained organoids were carefully mixed with unstained organoids and were seeded in three culture wells per condition. Standard culture medium was added, after which wells at culture initiation were imaged using a fluorescent microscope as well as after 24 h, to evaluate whether any consequential transfer occurred to adjacent unstained organoids.

### Flow cytometry—fluorescence intensity and durability

The fluorescence signal intensity and durability were only quantified for CMFDA, as it proved the most applicable of the labeling techniques studied. Organoids were stained with green CMFDA (5, 15, or 25 μM) and subsequently washed three times, after which the fluorescence intensities were determined by flow cytometry. Additionally, organoids stained with CMFDA (15 μM) were reseeded in Matrigel^®^ and cultured for up to 7 days. At each time point (day 0, 1, 2, 3, 4, and 7), organoids were harvested for analysis to assess the staining durability. This was performed once with three biologic replicates.

Organoids were harvested using Corning^®^ Cell Recovery Solution, after which they were enzymatically dissociated into single cells by incubation in TrypLE Express 1x (Thermo Fisher Scientific) for 20 min at 37 °C. Cells were subsequently stained with of eFlour™780 viability dye (1 μl) (Thermo Fisher Scientific) per 10^6^ cells/ml for 15 min, after which cells were washed in DPBS containing BSA (0.1%).

All samples were run on a BD FACS Canto™ II system (Becton Dickinson, Franklin Lakes, NJ, USA) and analyzed using BD FACSDiva software 8.0.1. PMT voltages were set manually by running samples of relevant cells. Compensation settings for eFlour™780 (APC-Cy7channel) and green CMFDA (FITC channel) were attained using the software’s automated compensation controls. The epithelial cell population was identified based on FSC-A and SSC-A, whereas FSC-H and FSC-A were correlated to identify singlets, after which eFlour™780/APC-Cy7-negative cells were isolated for ensuing analysis. Approximately 10,000 colonic epithelial cells were run per sample. The signal in unstained controls was used to quantify autofluorescence at baseline. Gating strategies are displayed in Additional file 2: Figure S1.

### Organoid forming efficiency following single cell seeding

Organoids were cultured in standard cultured medium for 7 days and subsequently enzymatically passaged into single cells using TrypLE express, as described above for flow cytometry. The single cells were subsequently stained with green CMFDA (5, 15, or 25 μM), after which they were washed and homogenized in diluted Matrigel^®^, supplemented with Jagged-1 (1 μM) (AnaSpec, Fremont, CA, USA) [23] and seeded in triplicates. Cells were cultured in standard medium (without supplementation of ROCK inhibitor) for a duration of 10 days. At day 10, the number of organoids was determined manually using an inverted digital light microscope. Six experiments were performed in total and included unstained DMSO (dimethylsulfoxid)-control samples as well as cells that briefly were treated with Triton™ X-100 (5%) (Sigma-Aldrich).

### PrestoBlue™ cell viability assay

Cell viability following labeling was assessed using the PrestoBlue™ cell viability assay (Thermo Fisher Scientific). Organoids were mechanically dissociated by brief pipetting and divided into five comparable samples (5, 15, or 25 μM of CMFDA, as well as a positive and negative control) and labeled in accordance with previously described staining protocol. Cells were seeded in Matrigel^®^ and cultured in standard culture medium for 24 h and 48 h. At each time point, the culture medium was replaced fresh medium supplemented with PrestoBlue™ cell viability reagent (10%) (Thermo Fisher Scientific). Organoids were incubated for 3–4 h after which 150 μl of the medium from each well was transferred to a 96-well plate (TPP, Trasadingen, Switzerland). Fluorescence was measured using a Synergy HT plate reader (BioTek Instruments, Winooski, VT, USA), excitation 530/25 emission 590/35. The positive control was very briefly treated with Triton™ X-100 (5%). In total, six experiments were performed in triplicates for each time point, and all viability data were compared to unstained DMSO-control samples.

### Hematoxylin and eosin staining and gene expression analysis of differentiation markers

Effects of staining with CMFDA on the stem cell differentiation capacity and the expression of marker genes were studied by comparing unstained and stained cells, cultured for 3 days in differentiation medium without Wnt3a, R-spondin 1, SB202190, and nicotinamide [19]. Gene expression levels in organoids cultured in standard proliferation medium were used to verify induction of differentiation. Organoids were harvested using Corning^®^ Cell Recovery Solution and lysed in PR1 buffer (Macherey-Nagel, Düren, Germany). RNA extraction was performed using a NucleoSpin^®^RNA purification kit (Macherey-Nagel), and reverse transcription was ensured using a Mastercycler^®^ (Eppendorf, Hamburg, Germany). All samples were run in three technical replicates on a LightCycler^®^ 480 (Roche, Basel, Switzerland) system, and six individual experiments were performed in total. The following lineage-specific differentiation markers were used: leucine-rich repeat-containing G-protein coupled receptor 5 (*LGR5*; intestinal stem cells), mucin 2 (*MUC2*; goblet cells), carbonic anhydrase II (*CAII*, mature enterocytes), and chromogranin A (*CHGA*; enteroendocrine cells). TATA-Box Binding Protein (*TBP*) was used as internal reference genes to normalize quantitative gene expression data. All primer sequences are listed in Additional file 1: Table S1.

Additionally, before and after differentiation, CMFDA-stained organoids were centrifuged and embedded into an artificial clot generated by adding a few drops of human plasma (produced in house) and bovine thrombin (CAS.9002-04-4, Merck, Darmstadt, Germany). Cells were then fixed with paraformaldehyde (4%) (Sigma-Aldrich) and embedded into paraffin. Slides were stained with hematoxylin-eosin before microscopic evaluation of cell distribution, nuclear features, and organoid morphology. Furthermore, organoids were stained with the cytokeratin 20 (CK20; FLEX monoclonal mouse anti-human cytokeratin 20, clone K 20.8, ready-to-use, cat.GA777, DAKO, Agilent Technologies), a general marker for colonic epithelial differentiation.

### Visualization of labeled and unlabeled cells in vitro using CLE

At days 0 and 3, stained organoids were visualized using an endoscope-based Pentax ISC-OU1000 system with a Pentax EC-3870 CIFK confocal laser endomicroscope (Pentax, Tokyo, Japan). This system is approved for clinical use and has a miniature confocal microscope integrated into the tip of a conventional endoscope. The tip of the endoscope was placed in the culture well within the Matrigel^®^ dome, and consecutive spatial planes were imaged. Unstained DMSO-samples were used to validate that no epithelial autofluorescence could be detected. Organoids derived from mT/mg mice expressing the tdTomato fluorescent protein were also imaged in vitro with CLE.

### Transplantation of intestinal epithelial cells into a DSS-model and ensuing imaging

Transplantation was performed in accordance with a previously published protocol [24], although with minor modifications. RAG2−/− mice (cat. B6.129S6-Rag2^tm1Fwa^N1, Taconic Biosciences, Rensselaer, NY, USA) were treated with dextran sodium sulfate (3.2%) (DSS; 36000–50,000 MW, colitis grade, MP Biomedicals, Santa Ana, CA, USA) in the drinking water for 5 days, and transplantations were performed 9 days after initiation of the DSS administration. Colonic organoids derived from mT/mG mice were released from Matrigel^®^ and mechanically dissociated into sheets of epithelial cells before incubation with CMFDA (15 μM) at 37 °C for 45 min followed by three washes. RAG2−/− mice were anesthetized by inhalation of isoflurane (2.5–3%) (cat. 055226, ScanVet Animal Health, Fredensborg, Denmark), and a suspension of organoid fragments from approximately 1000 colonic organoids resuspended in DPBS (300 μl) with Matrigel^®^ (5%) was infused into the colonic lumen using a syringe and thin flexible catheter. After infusion, the anal verge was glued for 3 h, and animals were sacrificed 24 h after transplantation. The timeline is depicted in Fig. 5b. Colons were harvested, and tdTomato- and CMFDA-positive regions were identified and imaged using a fluorescence dissecting microscope and with CLE.

### Statistical analysis

Statistical analyzes were performed using GraphPad Prism 8.0.0. Wilcoxon matched-pairs signed rank test was used to analyze cell viability, organoid forming efficiency, and gene expression data. Decline in fluorescence signal intensity determined by flow cytometry was analyzed by performing log_2_ transformation and subsequent linear regression. Results were considered statistically significant at *p* < 0.05.

## Results

### Staining outcome and homogeneity

**Fig. 1:**
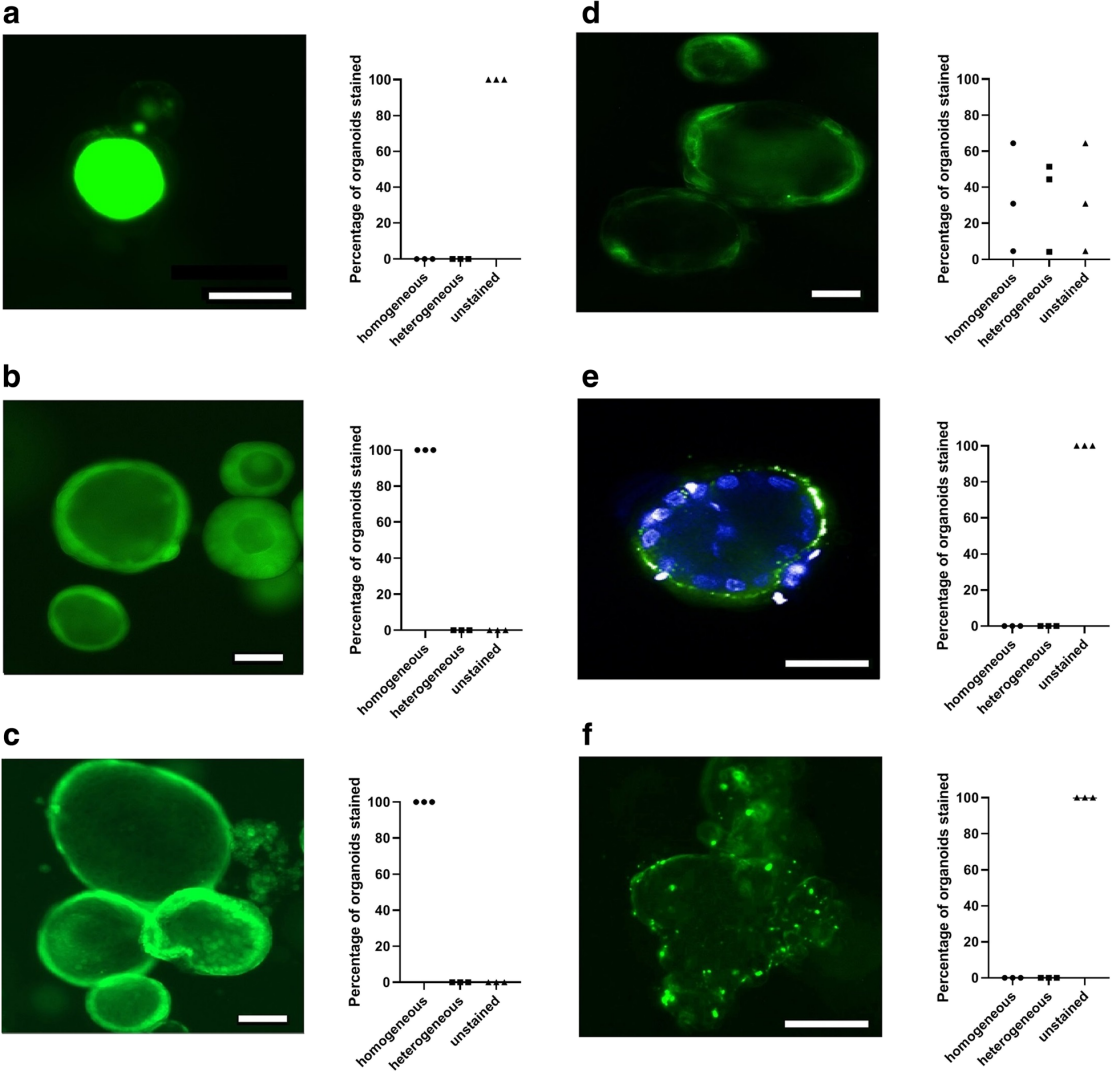
Staining of human colonic organoids. Fluorescent imaging of intestinal organoids stained with fluorescent dyes or nanoparticles, along with assessments of staining quality (homogeneous, heterogeneous or unstained). Standard fluorescent microscopy of organoids stained with **a** fluorescein, depicting accumulation in the organoid lumen, **b** an inert membrane permeable dye, **c** CMFDA or **d** a carbocyanine-based cytoplasmic membrane dye. **e** Confocal fluorescent imaging showing membrane-associated PLGA nanoparticles that have not been internalized into the cells. Nuclei are stained with Hoechst 33342. **f** Standard fluorescent imaging of intestinal organoids after attempted staining with quantum dots, depicting particle aggregation in proximity of the organoid. White scale bar, 100 μm

Fluorescein failed to stain any of the cells and merely accumulated in the organoid lumen (Fig. 1a), whereas CMFDA, the membrane permeable inert dye, as well as the carbocyanine-based cytoplasmic membrane dye, were easily internalized in the cells and effectively stained the organoids (Fig. 1b–d). Both the inert dye and CMFDA stained the organoids uniformly and appeared to stain all cells (Fig. 1b, c). In contrast, the carbocyanine-based cytoplasmic membrane dye (Fig. 1d) only stained a subset of the organoids homogeneously (median = 31%, ICR = 5–65%), whereas a comparable fraction of organoids were either heterogeneously stained (median = 44%, ICR = 4–52%) with only a subset of cells labeled or not stained at all (median = 25%, ICR = 4–65%).

Attempted staining of organoids or single cells with PLGA nanoparticles in suspension resulted in some particles (uncoated and coated alike) becoming membrane-associated around the organoid periphery, but without obvious signs of actual internalization of the nanoparticles (Fig. 1e). Similarly, no staining was achieved when the PLGA nanoparticles (coated or uncoated) were added to the Matrigel^®^ solution before polymerization or when added to the culture medium, as the particles aggregated around the organoids or were trapped in the periphery of the Matrigel^®^ dome.

Labeling of whole organoids or single cells using quantum dots similarly failed, as particles merely gathered near the cells or organoids and were not internalized (Fig. 1f).

### Intercellular transfer of the dyes

**Fig. 2:**
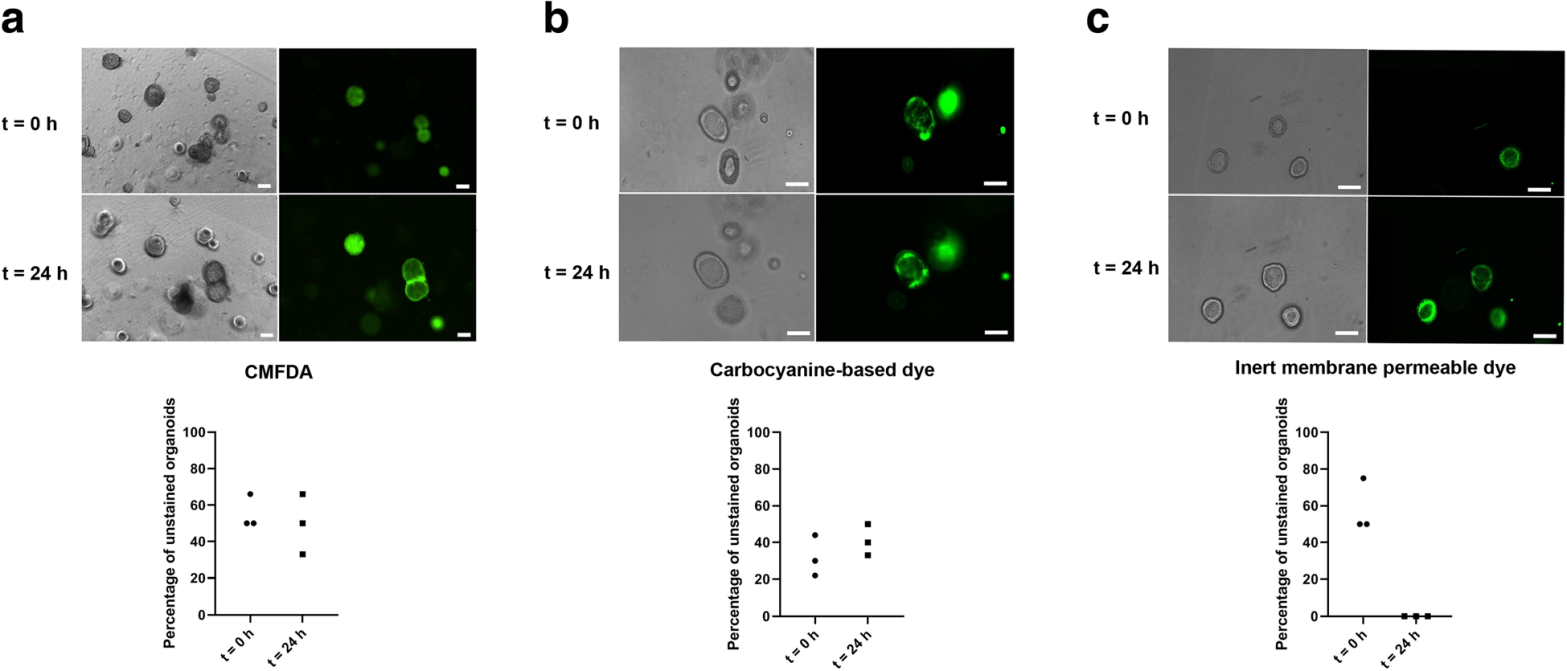
Intercellular dye transfer. Fluorescent and brightfield imaging of co-cultures of unstained and stained colonic organoids, along with quantitative assessment of dye transfer. Organoids were stained with **a** CMFDA, **b** carbocyanine-based cytoplasmic membrane dye or with **c** an inert membrane permeable dye. Images were taken at initiation of the co-cultures as well as 24 h later. White scale bar, 50 μm

No transfer of dye was observed when organoids stained with CMFDA or carbocyanine-based cytoplasmic membrane dye were co-cultured with unstained organoids for 24 h (Fig. 2a, b). The median fraction of unstained organoids at culture initiation was 30% (ICR = 22–44%) and 50% (ICR = 50–66%) for CMFDA and the carbocyanine-based cytoplasmic membrane dye, respectively. After 24 h, the fractions were maintained at comparable levels (CMFDA median: 40%, ICR = 33–50% and carbocyanine-based dye 50%, ICR = 33–66%). However, rapid transfer of the membrane permeable inert dye from stained to unstained organoids was observed, making these two populations indistinguishable after approximately 1 h (Fig. 2c), with no unstained organoids remaining.

### Fluorescence signal intensity and durability

**Fig. 3:**
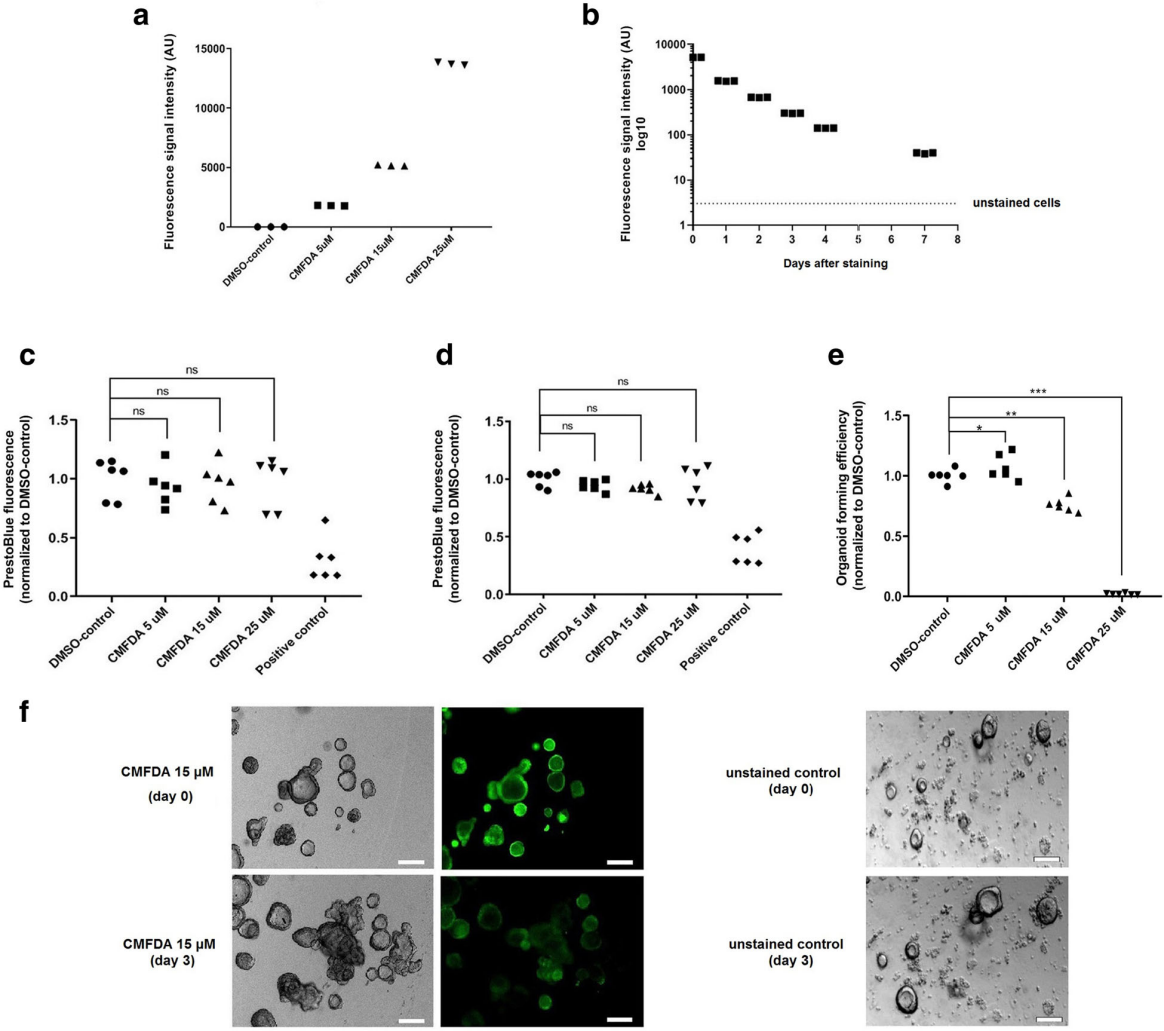
Organoid forming efficiency, viability, and fluorescence signal durability. **a** Fluorescence signal intensity immediately after staining with CMFDA (5–25 μM), determined by flow cytometry. **b** Decline in fluorescence signal intensity (15 μM CMFDA) over time. **c**, **d** PrestoBlue^TM^ viability assay performed consecutively **c** 24 h and **d** 48 h after staining with CMFDA (5–25 μM). Arbitrary unit (AU). No statistically significant (ns) difference in viability was detected. **e** Organoid forming efficiency determined 10 days following single-cell seeding of CMFDA-stained (5–25 μM) and unstained cells. All values were normalized to the mean of the unstained DMSO-control. A statistically significant increase was observed with 5 μM (*) of CMFDA (*p* = 0.03) (CMFDA median = 1.034% ICR = 0.998–1.186%, DMSO median = 1.003% ICR = 0.976–1.024%). A drastic and statistically significant reduction of the organoid forming efficiency was observed with increasing concentrations of with CMFDA 15 μM (**) (median = 0.75% ICR = 0.710–0.797%) and 25 μM (***) (median = 0.017% ICR = 0.013–0.024%). **f** Brightfield and fluorescent images of organoids at day 0 and day 3 after staining with CMFDA (15 μM), along with unstained controls, demonstrating maintained growth capacity. White scale bar, 100 μm

Since CMFDA was the only dye that efficiently stained the organoids without transferring to neighbouring unstained cells, further studies were only carried out for this fluorophore. The CMFDA-derived fluorescent signal intensity increased exponentially with increasing concentration (5–25 μM, Fig. 3a), and 99% of cells were CMFDA-positive, as determined by flow cytometry.

The fluorescence signal intensity of intestinal epithelial cells stained with of CMFDA (15 μM) was reduced by *t*_½_ of 0.99 days (*r*^2^ = 0.96, Fig. 3b). The signal intensity approached levels in unstained cells approximately 4–7 days after staining.

### Cell viability and organoid forming capacity

The PrestoBlue™ viability assay did not reveal any statistically significant reduction in cell viability 24 h or 48 h after staining of organoid fragments with 5–25 μM of CMFDA (Fig. 3c, d). The organoid forming capacity following single-cell seeding was notably affected with increasing concentration of CMFDA (Fig. 3e). No atypical growth behavior of CMFDA-labeled organoids (cultured from fragments) was observed, and organoids visibly continued to grow (Fig. 3f). Some events of organoids dissociation were observed after staining but no clear concentration-dependent trend was observed, and it was not sufficiently prevalent to be detected by the viability assay.

### Cell differentiation and gene expression analysis

**Fig. 4:**
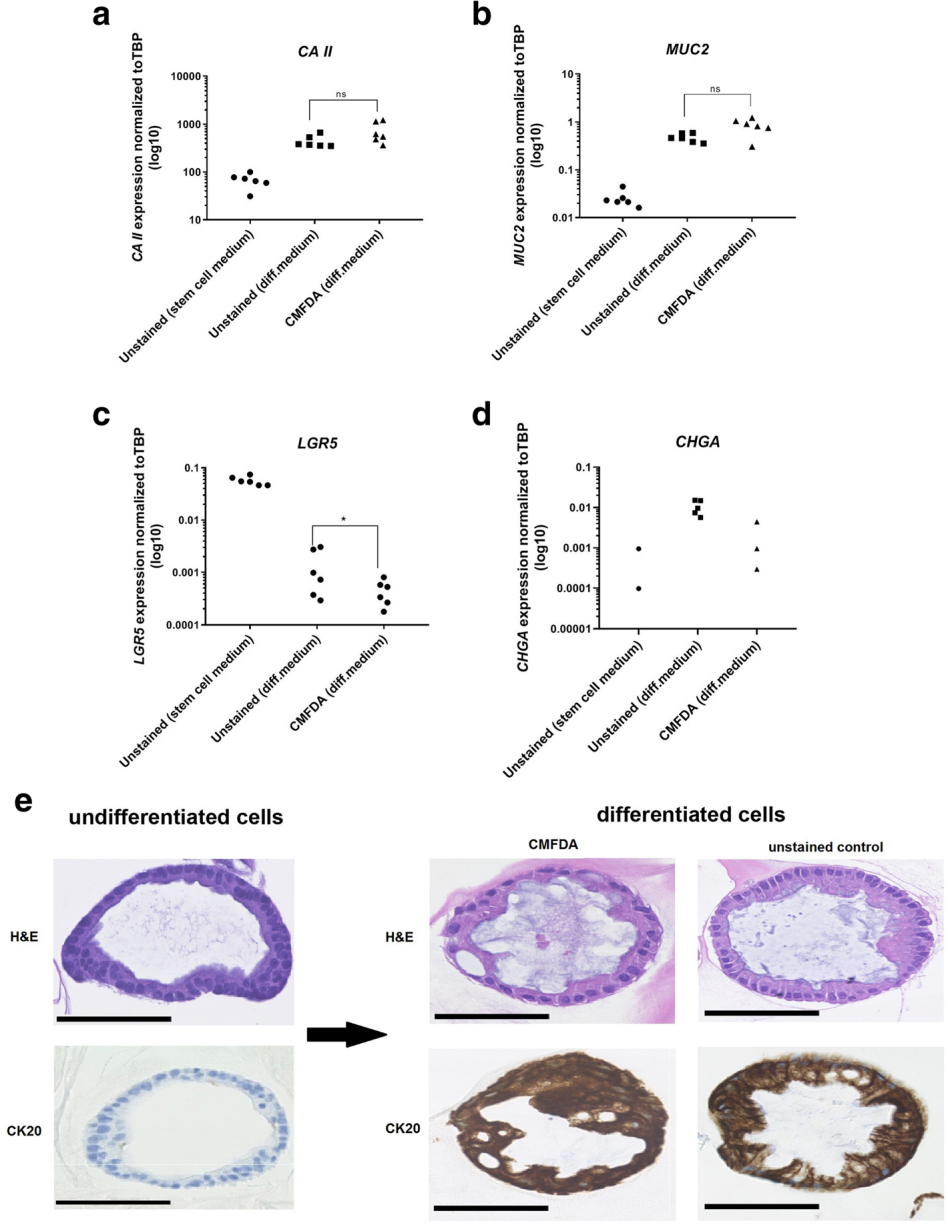
Cell differentiation analysis. **a–d** Gene expression analysis of lineage specific differentiation marker genes in unstained (DMSO) and CMFDA-stained cells following 3 days of differentiation. Expression levels in cells cultured in standard culture medium are similarly depicted. Data are presented as Ct-values of target genes normalized to the housekeeping gene (*TBP*). **a** *CAII* (enterocytes), **b** *MUC2* (goblet cells), **c** *LGR5* (intestinal stem cells), and **d** *CHGA* (enteroendocrine cells). A significant reduction in the expression of *LGR5* (*) in CMFDA-stained cells was detected following differentiation. No statistically significant (ns) difference was observed in the expression levels *CA II* or *MUC2*. The expression of *CHGA* could be detected in a few samples only (both stained and unstained) but indicated increased expression levels following differentiation. **e** Staining with hematoxylin-eosin and cytokeratin 20 (CK20) of unstained and CMFDA-labeled organoids before and after differentiation, confirming presence of absorptive colonocytes along with secretory goblet cells in both conditions. Cell nuclei are spherical and basally located, consistent with more differentiation phenotype. Strong reaction for CK20 after differentiation. Hence, no signs that the differentiation capacity is affected by CMFDA. Black scale bar, 100 μm

Upon induction of differentiation, a significant increase in the expression of *CA II* and *MUC2* was observed, with no difference between unstained and CMFDA-stained cells after differentiation (Fig. 4a, b). In both CMFDA-stained cells and DMSO-controls, a statistically significant decrease was detected in the *LGR5* expression (*p* < 0.05) upon induction of differentiation (Fig. 4c). The median expression level of *LGR5* after differentiation was 0.8%, ICR = 0.5–1.2% (CMFDA) and 1.6%, ICR = 0.7–4.9% (DMSO-controls) of levels in unstained cells before differentiation, and the difference proved to be statistically significant (*p* < 0.05).

The expression of CHGA proved to be undetectable in several samples (both stained and unstained cells, Fig. 4d), but with a clear trend towards increased expression levels following induction of differentiation.

H&E staining and subsequent microscopic evaluation of CMFDA-labeled organoids along with unstained DMSO-control organoids revealed increasingly differentiated cell morphology with simple columnar epithelium in both conditions. Absorptive colonocytes as well as goblet cells with mucus filled vacuoles could easily be identified, with a rich luminal accumulation of mucus. Nuclei were basally located and primarily spherical in shape (Fig. 4e). A very strong positive reacted for CK20 was detected after differentiation in both conditions (Fig. 4e).

### Imaging of labeled cells in vitro with CLE

**Fig. 5:**
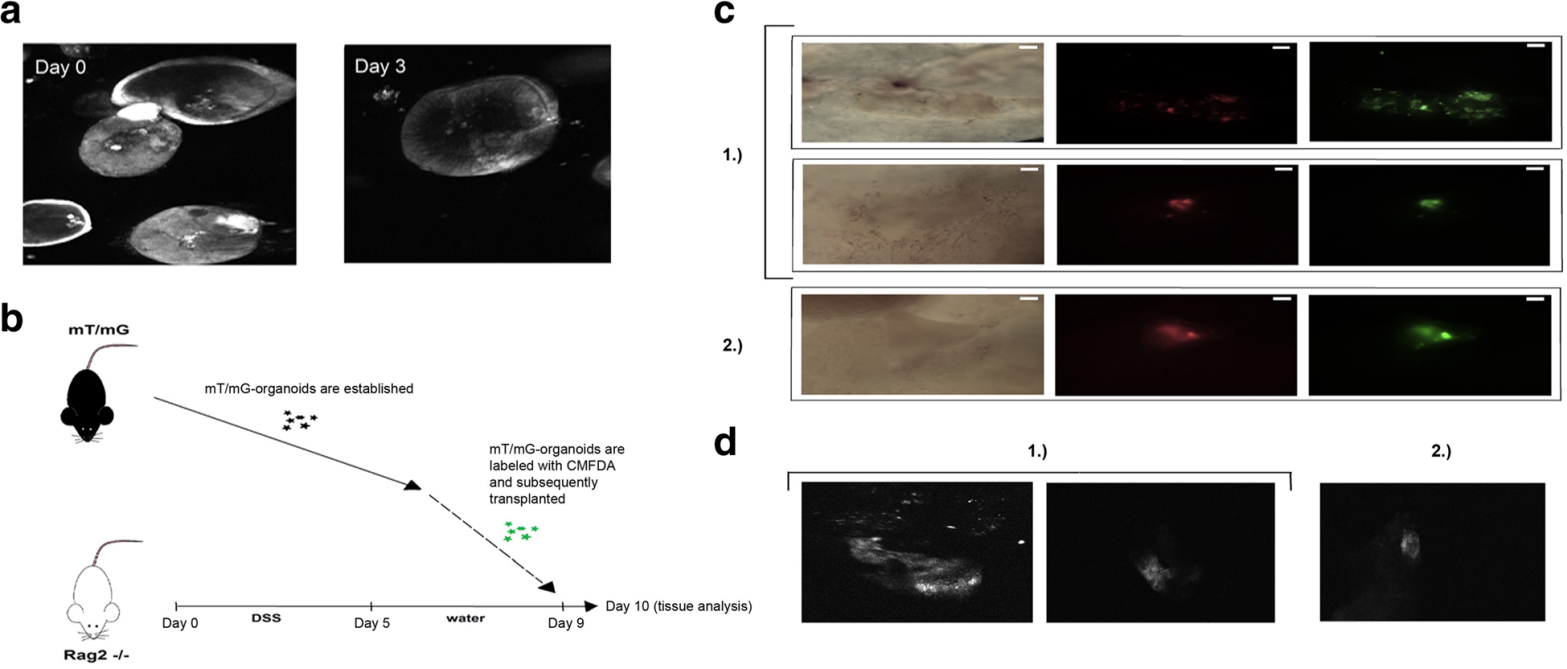
Imaging in vitro and ex vivo with fluorescent microscopy and CLE. **a** Intestinal organoids labeled with CMFDA (15 μM) and imaged in vitro on day 0 and day 3 with endoscope-based CLE. Standard gain settings were used. Approximately × 1000 magnification. No scale bar available. Image diameter approximately 100–200 μm. **b** Rag2−/− mice were treated with dextran sodium sulfate (DSS) for 5 days. DSS was administered for 5 days, and 4 days later (on day 9), colonic organoids derived from mT/mG mice were labeled with CMFDA and transplanted (by colonic infusion) into the Rag2−/− mice. The recipient mice were sacrificed the subsequent day (day 10), and the colonic tissue was harvested for analysis. **c** Brightfield and fluorescent images of colonic tissue mucosa from two different recipient mice (1 and 2) and three separate colonic regions depicting retained mT/mG (red) and CMFDA (green) positive cells. White scale bar, 1 mm. **d** Fluorescent images of the corresponding colonic regions (recipient mouse 1 and 2) taken with an endoscope-based CLE. No scale bar available

Intestinal organoids could effectively be identified with CLE for at least 3 days following staining with CMFDA (Fig. 5a). In addition to organoid identification, rudimentary morphology and 3D structure, as well as occasional budding, could be distinguished. No autofluorescence from unstained organoids was detected.

### Ex vivo imaging of CMFDA-labeled cells

To assess whether labeled cells could be identified ex vivo, transplantation of CMFDA-labeled cells was performed using a murine DSS-model [3, 5]. Twenty-four hours after transplantation, fluorescent regions of the colon (three separate regions in two different recipient mice) were identified ex vivo as both CMFDA- and TdTomato-positive using a fluorescence dissecting microscope (Fig. 5c). The corresponding fluorescent signal could similarly be detected with CLE by placing the tip in contact with the mucosa (Fig. 5d). No fluorescent signal was detected from the surrounding regions and imaging of mT/mG organoids confirmed that the tdTomato signal was not readily detected by the CLE, unlike the CMFDA signal (Additional file 3: Figure S2).

## Discussion

For efficient dye-based fluorescent tracing in vivo, the dye must efficiently and homogenously stain the cells without affecting their viability or function, while also transferring to daughter cells. Additionally, it is important that the stain does not transfer to adjacent resident cells, as this would render it useless for tracing purposes. Hence, fluorescein, the freely membrane permeable dye and the carbocyanine-based dye proved to be ineligible, whereas CMFDA appeared to display the necessary qualities for this type of tracing application. Once it passes through the plasma membrane, it becomes membrane impermeable through a supposed glutathione-mediated process, and a subsequent reaction with thiol groups of intracellular proteins. CMFDA staining did not appear to significantly affect organoid functions or viability, at least not when staining organoid fragments. In fact, organoids derived from fragments continued to grow and maintained normal viability following CMFDA staining, as indicated by the PrestoBlue™ viability data. However, a concentration-dependent effect was observed on the organoid forming efficiency of single cells, and it has been reported in epithelial cancer cell lines that CMFDA can affect the mechanical properties of single cells [25]. The effect was notably less pronounced when using CMFDA at a concentration of 15 μM, and this concentration remained applicable for tracing purposes. Intestinal stem cell transplantation is performed with organoid fragments instead of single cells and we therefore do not expect CMFDA to affect the regenerative capacity of organoid fragments. Interestingly, in a recent human skin wound healing assay, wound healing was maintained despite consecutive staining with CMFDA [26].

CMFDA staining did not appear to restrain cell differentiation into absorptive or secretory lineage, and cells clearly attained a more differentiated phenotype, comparable to unstained cells, after induction of differentiation. Nonetheless, induction of differentiation in vitro by removal of critical niche factors occurs at the expense of organoid maintenance, which leaves a narrow window to study cell differentiation [27]. To quantify the stem cell progeny and to thoroughly assess any effects of CMFDA will likely require transplantation, as additional cell differentiation can be achieved in vivo [2, 3, 28, 29]. The difference in *LGR5* expression in vitro following differentiation was relatively modest and did not seem to affect organoid growth but could theoretically be at least partially responsible for the effect on single-cell organoid forming capacity. The expression of *CHGA* could be detected only in a few samples (both stained and unstained). This observation is not surprising, as enteroendocrine cells only comprise < 1% of the total number of cells of the intestinal epithelium.

We observed a fluorescence signal retention of roughly 4–7 days in CMFDA-stained cells, with a decline in fluorescence over time most likely by means of dilution as cells divided. Nonetheless, CMFDA-stained cells could efficiently be identified using CLE in vitro for at least 3 days. Albeit the CMFDA-signal intensity depends on a combination of factors (e.g., the proliferation rate, the intracellular amount of CMFDA, as well as the number of cells), CMFDA-based tracing may be applicable for even longer than 3 days in vivo, as human colonic stem cells have been found to be slow cycling [4].

In an attempt to increase the longitudinal duration of fluorescence-based tracing, we included the dye-loaded PLGA nanoparticles and quantum dots in our investigation. Cell uptake of quantum dots is dependent on conjugation with targeting ligands such as peptides, arginine-glycine-aspartate (RGD), transactivator of transcription (TAT), antibodies, or small molecules [30–32]. Although targeting moieties is an option [33, 34], cell uptake of PLGA particles is generally regulated by other modifiable factors (e.g., particles size [35], surface charge [36–38], and incubation time [39]). Nonetheless, primary intestinal epithelial cells were unable to internalize PLGA nanoparticles or quantum dots. This despite that the PLL-coated PLGA particles had a comparably positive ζ-potential to what has been used to internalize particles in other cell types [16, 21]. Additionally, the particles were only slightly larger than 100 nm, which in Caco-2 cells have been demonstrated to be more effectively internalized than particles with a larger diameter [40]. However, there are conflicting reports on the uptake of PLGA nanoparticles, even in Caco-2 cells, with one study reporting limited internalization even after several hours of incubation [39].

The targeting mechanism used in the *Qtracker*™ *cell labeling kit* is undisclosed and proprietary, which makes troubleshooting difficult, but it is possible that efficient internalization may be achieved using alternative targeting methods. Similarly, uptake of PLGA particles can potentially be accomplished using other formulations or coating strategies. However, the fact that we were unable to internalize any of the nanoparticles suggests that primary intestinal epithelial cells are not as readily labeled as other types of cells (e.g., mesenchymal stem cells or cancer cell lines). Additionally, particle uptake is presumably made more difficult by the prevalent culture techniques and inherent properties of intestinal epithelial cells. When culturing intestinal organoids in Matrigel^®^, they spontaneously form spherical polarized structures (i.e., organoids), in which the basal surface of the cells faces the surroundings, while the apical side is oriented towards the sealed off lumen. This presumably reduces the ability of the cells to take up exogenous nanoparticles by means of endocytosis, as this mainly occurs from the apical side. Internalization is made even more difficult by the fact that organoids require cell-matrix interaction, and that Matrigel^®^ acts as a physical diffusion barrier. To circumvent these obstacles, we attempted to stain both single cells and organoid clusters while in suspension, yet without improving cell uptake. Prolongation of the time of incubation with nanoparticles to increase uptake is, however, not feasible due to the high dependency of cell-basal membrane interaction for intestinal organoid survival.

Intestinal epithelial cells inaptitude to internalize nanoparticles also has consequences for alternative imaging modalities such as MRI, as it necessitates internalization of a contrast agent (e.g., iron oxide or gadolinium), which at least in non-phagocytic cells requires the use of nanoparticles like PLGA particles or specific coatings [41].

Our transplantation experiments suggest that it indeed is possible to detect retained intestinal epithelial cells using a common fluorescent dye and CLE, but the experiments were qualitative rather than quantitative in design. It still remains unclear how to best define the engraftment efficiency in this setting, as transplantation commonly is performed with large numbers of organoid fragments without knowing the exact number of cells. Similarly, it is not clear how or when to best quantify the number of engrafted cells, but we believe that our tracing strategy allows for evaluation of engraftment efficiency along with factors that can affect the outcome, such as severity of ulceration and inflammation, age of recipient, and the applied cell delivery method.

## Conclusion

It is highly feasible to trace transplanted human intestinal organoids using fluorescent dyes (e.g., CMFDA) in combination with clinically approved CLE. CMFDA did not significantly affect organoid viability or growth, and the stem cell differentiation capacity remained intact. The approach may, although limited to short-term tracing, allow confirmation of implantation at the intestinal target site following transplantation. This, in turn, will permit evaluation of engraftment efficiency, which is crucial for further development of this type of novel treatment strategy.

## Additional files

Additional file 1: **Table S1.** Nucleotide sequences used for gene expression analysis of differentiation markers. (DOCX 15 kb)

Additional file 2: **Figure S1.** Gating strategies for flow cytometry analysis. (a) The relevant population of colonic epithelial cells was identified based on FSC-A and SSC-A. (b) Single cells were isolated based on the correlation between FSC-H and FSC-A. (c) eFlour™780/APC-Cy7-negative cells were isolated, thereby excluding dead cells from the subsequent analysis. (d) The CMFDA-derived FITC signal intensity was subsequently quantified. (JPG 486 kb)

Additional file 3: **Figure S2.** CLE imaging of mT/mG organoids in vitro. Murine mT/mG organoids could not be made out in vitro (left) unless maximizing the image gain (right) and thereby drastically reducing the image quality. (JPG 844 kb)

## Abbreviations

CA II: Carbonic anhydrase II
CHGA: Chromogranin A
CK20: Cytokeratin 20
CLE: Confocal laser endomicroscopy
CMFDA: 5-Chloromethylfluorescein diacetate
DMEM: Dulbecco’s modified Eagle medium
DMSO: Dimethylsulfoxid
DPBS: Dulbecco’s phosphate-buffered saline
EDTA: Ethylenediaminetetraacetic acid
EGF: Epidermal growth factor
H&E: Hematoxylin-eosin
LGR5: Leucine-rich repeat-containing G-protein coupled receptor 5
MRI: Magnetic resonance imaging
MUC2: Mucin 2
PCR: Polymerase chain reaction
PLGA: Polylactic-co-glycolytic acid
PLL: Poly-l-lysine
RT-qPCR: Reverse transcriptase quantitative PCR
TBP: TATA-box binding protein
