# Supplementary material for: Fluorescence-based tracing of transplanted intestinal epithelial cells using confocal laser endomicroscopy
Source: Stem Cell Res Ther. 2019 May 27;10:148. doi: 10.1186/s13287-019-1246-5 (PMC6537188; doi:10.1186/s13287-019-1246-5)
Supplement: Supplementary file 1 — Table S1. Nucleotide sequences used for gene expression analysis of differentiation markers. (DOCX 15 kb) [file 13287_2019_1246_MOESM1_ESM.docx]

**Table S1 - Nucleotide sequences used for gene expression analysis of differentiation markers**

| **Primer** | **Nucleotide sequence 5’- to –’3** |
| --- | --- |
| *LGR5* - fwd | GAGTTACGTCTTGCGGGAAAC |
| *LGR5* - rev | TGGGTACGTGTCTTAGCTGATTA |
| *MUC2* - fwd | AGGATGACACCATCTACCTCAC |
| *MUC2* - rev | CATCGC TCTTCTCAATGAGCA |
| *CA* *II* - fwd | GGGTACGGCAAACACAACG |
| *CA* *II* - rev | GGCTGTATGAGTGTCGATGTC |
| *CHGA* - fwd | TAAAGGGGATACCGAGGTGATG |
| *CHGA* - rev | TCGGAGTGTCTCAAAACATTCC |
| *TBP* - fwd | CACGAACCACGGCACTGATT |
| *TBP* - rev | TTTTCTTGCTGCCAGTCTGGAC |

Leucine-rich repeat-containing G-protein coupled receptor 5 (*LGR5*), Mucin 2 (*MUC2*), Carbonic Anhydrase II (*CA II*), Chromogranin A (*CHGA*) and TATA-Box Binding Protein (*TBP*).
