# Supplementary figures and images for: Fluorescence-based tracing of transplanted intestinal epithelial cells using confocal laser endomicroscopy
Source: Stem Cell Res Ther. 2019 May 27;10:148. doi: 10.1186/s13287-019-1246-5 (PMC6537188; doi:10.1186/s13287-019-1246-5)

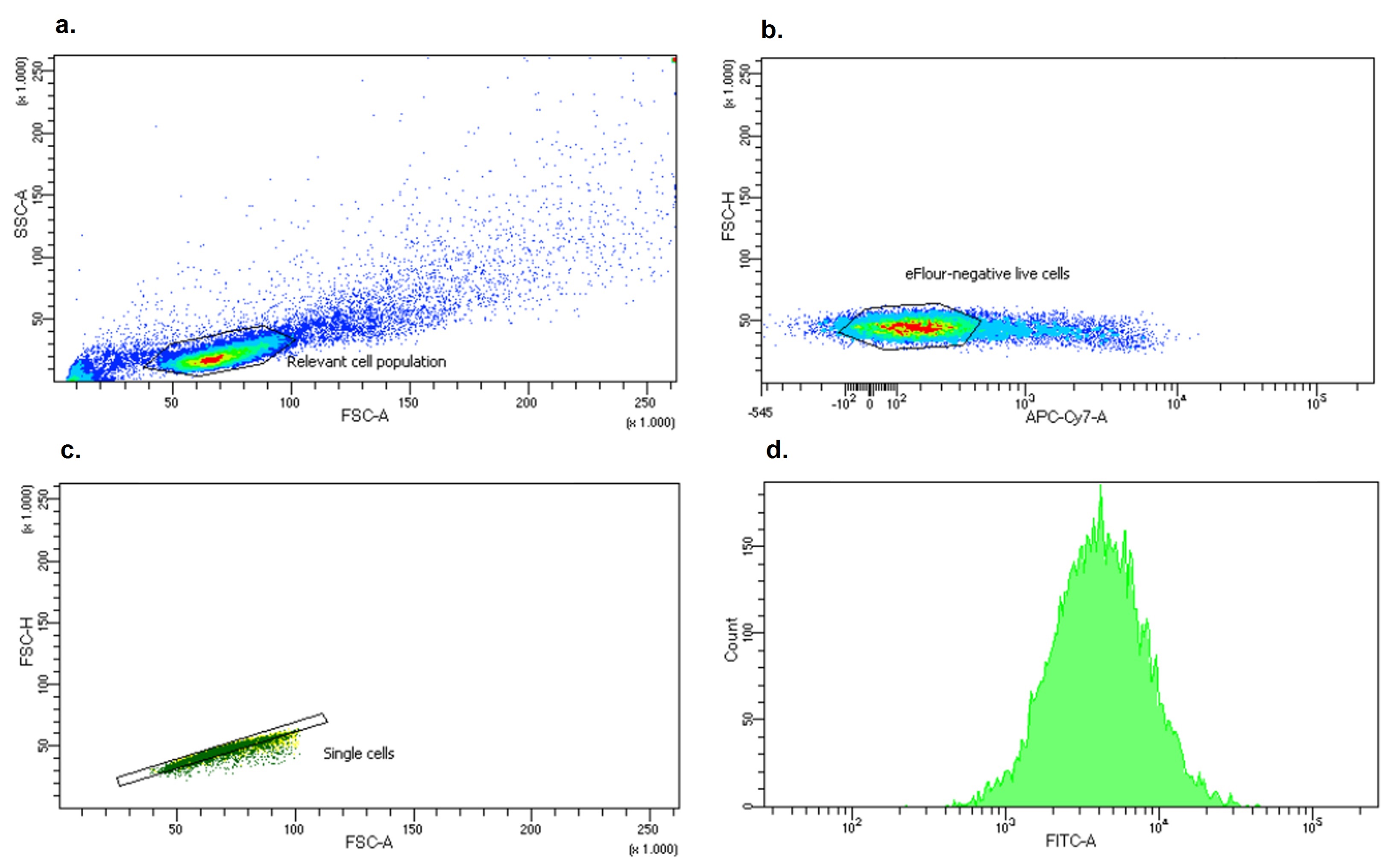

Supplement: Supplementary file 2 — Figure S1. Gating strategies for flow cytometry analysis. (a) The relevant population of colonic epithelial cells was identified based on FSC-A and SSC-A. (b) Single cells were isolated based on the correlation between FSC-H and FSC-A. (c) eFlour™780/APC-Cy7-negative cells were isolated, thereby excluding dead cells from the subsequent analysis. (d) The CMFDA-derived FITC signal intensity was subsequently quantified. (JPG 486 kb) [file 13287_2019_1246_MOESM2_ESM.jpg]

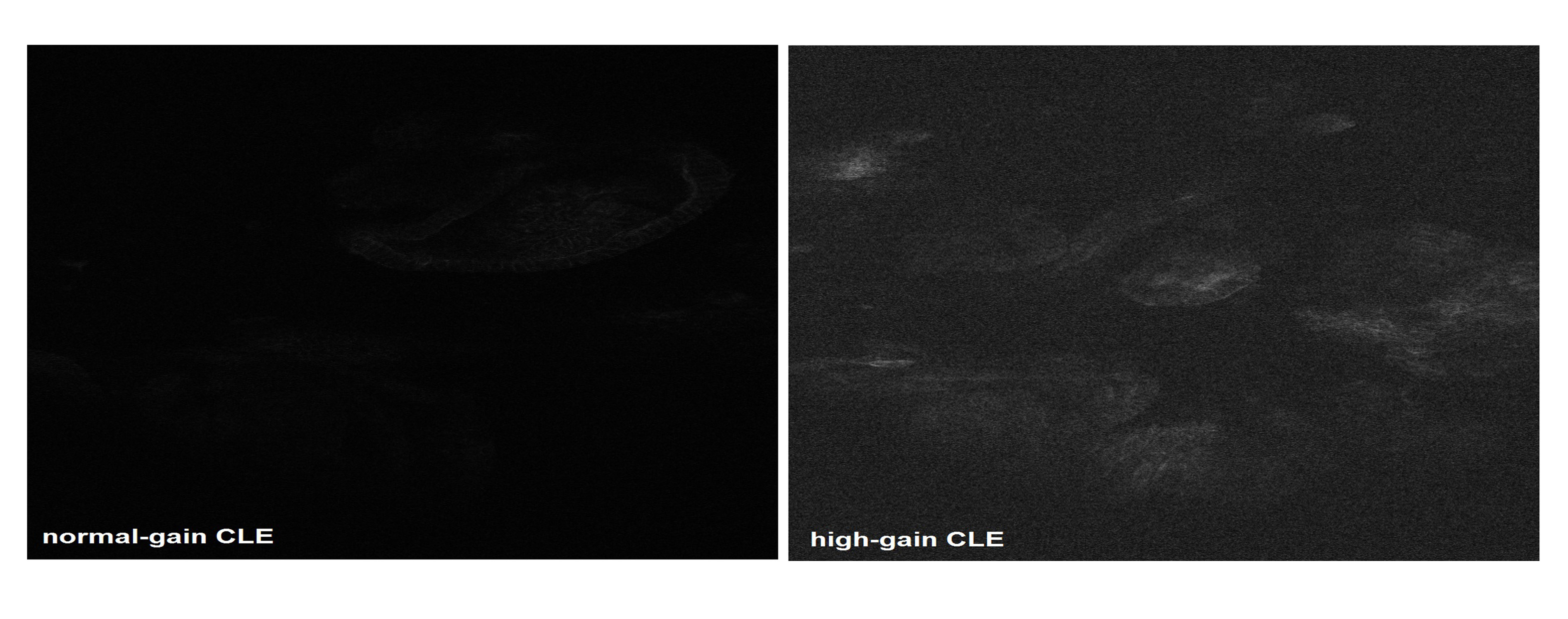

Supplement: Supplementary file 3 — Figure S2. CLE imaging of mT/mG organoids in vitro. Murine mT/mG organoids could not be made out in vitro (left) unless maximizing the image gain (right) and thereby drastically reducing the image quality. (JPG 844 kb) [file 13287_2019_1246_MOESM3_ESM.jpg]
